# Impact of COVID-19 on Mental Health in Adolescents: A Systematic Review

**DOI:** 10.3390/ijerph18052470

**Published:** 2021-03-03

**Authors:** Elizabeth A. K. Jones, Amal K. Mitra, Azad R. Bhuiyan

**Affiliations:** School of Public Health, College of Health Sciences, Jackson State University, Jackson, MS 39213, USA; eakjones@yahoo.com (E.A.K.J.); azad.r.bhuiyan@jsums.edu (A.R.B.)

**Keywords:** mental health, mental illness, mental disorder, psychiatric illness, anxiety, depression, COVID 19, coronavirus, 2019-ncov, sars-cov-2, cov-19, adolescent, teenagers

## Abstract

Due to lack of sufficient data on the psychological toll of the COVID-19 pandemic on adolescent mental health, this systematic analysis aims to evaluate the impact of the pandemic on adolescent mental health. This study follows the PRISMA guidelines for systematic reviews of 16 quantitative studies conducted in 2019–2021 with 40,076 participants. Globally, adolescents of varying backgrounds experience higher rates of anxiety, depression, and stress due to the pandemic. Secondly, adolescents also have a higher frequency of using alcohol and cannabis during the COVID-19 pandemic. However, social support, positive coping skills, home quarantining, and parent–child discussions seem to positively impact adolescent mental health during this period of crisis. Whether in the United States or abroad, the COVID-19 pandemic has impacted adolescent mental health. Therefore, it is important to seek and to use all of the available resources and therapies to help adolescents mediate the adjustments caused by the pandemic.

## 1. Introduction

The COVID-19 pandemic has created a havoc across the world, which has resulted in over 2 million deaths, globally and forced billions into isolation due to stay at home orders [1]. As a result of social isolation, and the constant concern of infectivity, mental health consequences that are associated with the COVID-19 crisis are monumental [2,3]. However, researchers are focusing more on the mental health impact of this rapidly evolving global crisis in the elderly population [4]. There has been very little attention to the psychological toll of COVID-19 on adolescent mental health [5].

The psychological toll of COVID-19 among adolescents should be a focal point in COVID-19 research due to the severe and enduring impact of mental health, which leads to poor mental health outcomes and to poor physical health outcomes, such as the development of cardiovascular diseases [6,7]. The mental toll of this impact serves as a greater challenge for adolescents because this age range (characterized as young people between the ages of 13–17) lack the psychological capabilities of resilience and coping and the physiological development of adults [6,8]. The mental health challenges of adolescents are even greater among adolescents with pre-existing mental health conditions during periods of crisis, which may be a result of isolation, feelings of uncertainty, lack of daily routines, lack of access to health services provided through schools, and lack of advanced emotional development [9,10].

Globally, 10–20% of adolescents suffer from mental health conditions [11]. This statistic is likely to be affected by the vulnerability of adolescents during the COVID-19 pandemic [12]. Due to the negative outcomes associated with poor mental health statuses among adolescents, such as suicide, behavior problems, and emotional distress and the need for quality research to build resilience and to reduce anxiety among adolescents, it is imperative to review the impact of COVID-19 on adolescent health in the United States and abroad in order to understand the global state of adolescent mental health and to provide strategies that prevent poor mental health outcomes, such as anxiety and depression, presently and in the future [13].

The aim of this systematic review is to assess the impact of the COVID-19 pandemic on adolescent mental health. Distinctively, the objectives of this review are: (1) To identity the state of adolescent mental health, globally; and (2) To provide quality research that will provide insight into strategies that can be used to address poor mental health outcomes of adolescents.

## 2. Materials and Methods

The systematic review included studies by following the PRISMA guidelines [11]. The study focused on published original quantitative studies on mental health issues in adolescents due to COVID-19. The inclusion and exclusion of the review are mentioned in Table 1. 

### 2.1. Search Guidelines

The primary search engines that were used to identify articles included EBSCOhost, MEDLINE, APA PsychoInfo, APA Psych articles, Socindex, and CINAHL. All the investigators were involved in the search process. The studies were chosen for the review based on inclusion criteria, such as (1) articles being written in English; (2) being quantitative studies; (3) being scholarly papers; (4) using human participants between the ages of 13–17; (5) being associated with mental health illnesses; and (6) being associated with COVID-19. The search was performed on 27 January 2021. The time limit for the studies was from 2019–2021. The search string is mentioned in Table 2.

### 2.2. Screening Guidelines

The Preferred Reporting Items for Systematic Reviews and Meta-Analysis (PRISMA) guidelines (2009) was used as a guide to record the review process [14]. Selected abstracts were reviewed to ensure their eligibility for inclusion. Full text articles of eligible abstracts were retrieved and assessed on whether they answered the research questions and fulfilled the inclusion criteria. Studies were included if consensus was reached by the three researchers.

**Figure 1:**
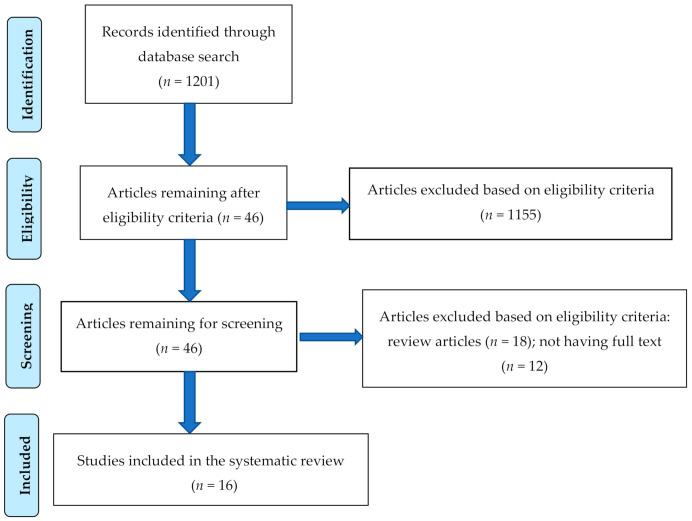
PRISMA flow chart to illustrate the article search and the inclusion process.

Research Information System (RIS) formatted references were exported from the databases, where studies were automatically screened based on the inclusion criteria and then imported into CADIMA. The 46 studies that were imported in CADIMA were accessed based on title and abstracts. The researchers (Jones, Mitra and Bhuiyan) accessed the studies two times before discussing if the studies should be chosen for full text review. Conflicts were managed by group discussions between all three of the researchers of this study. After the initial discussion, all of the researchers agreed that 46 studies should be selected for further screening using the inclusion criteria. During this second phase of screening for having articles with full text and for excluding review articles, the three researchers again screened the 46 articles two times independently. Conflicts were managed by group discussions. After discussion, 30 more articles were excluded because they did not meet the inclusion criteria, and 16 articles were selected to be included in the systematic review. The PRISMA flow chart (Figure 1) exhibits the search and inclusion process for the systematic review. 

### 2.3. Quality Appraisal 

Studies were appraised for quality using CADIMA. Through CADIMA, standards for the critical appraisal and the rating scale of the studies were defined. We followed the critical appraisal tools for systematic reviews developed by the University of Adelaide, South Australia [15]. A rating scale from 0 to 4 was based on the following criteria: (1) Study design—cross-sectional, case–control, or cohort study = 1, otherwise = 0; (2) Sample size—large = 1, small = 0; (3) Use of standardized instrument(s) for data collection, such as assessment of mental health by using Patient Health Questionnaire-9 (PHQ-9) or Diagnostic Manual of Mental Disorders-IV (DSM-IV) = 1, not specific = 0; and (4) Selection of participants—random selection or lack of bias = 1, non-random sample or convenience sample or presence of bias = 0 point. Based on the above-mentioned criteria, the three researchers rated each of the 16 studies independently from a range of 0 to 4. Due to having no major inter-observer variations in the evaluation of the quality of the studies, an average of the three scores was presented in Table 3. 

## 3. Results

A summary of the methodology, characteristics of findings, impact of COVID-19 on mental health in adolescents, quality appraisal and the countries of the studies are presented in Table 3. Of the 16 studies reviewed, 7 were conducted in China, 2 in the United States, 2 in Canada, and 1 each in Denmark, Germany, Japan, the Philippines, and the United Kingdom. All of the 16 studies utilized a quantitative methodology [16,17,18,19,20,21,22,23,24,25,26,27,28,29,30,31]. Among the studies, 12 (75%) were conducted online and 4 (25%) did not clearly report the study format. The studies were conducted from February 2020–May 2020, with six studies not reporting the date of data collection. Ten studies (62.5%) were conducted among adolescents only, three studies (18.8%) were conducted among children and adolescents, one study (6.3%) was conducted among adolescents and parents, and two studies (12.5%) were conducted among adults, young adults, and adolescents. In this study, we evaluated the data of adolescents only. 

The total sample size used in the studies varied from 102 to 9554, having a median sample size of 1054 (1st Quartile = 693 and 3rd Quartile = 3254); 10 out of 16 (63%) had sample sizes of more than 1000. In terms of standardized tools, nine studies (56%) utilized standardized tools, and seven studies (44%) did not use standardized tools. 

An average score of 4 out of 4, meant excellent in seven (44%), 2–3, meant moderate in seven (44%), and 0–1, meant poor in two (12%) studies. 

### 3.1. Anxiety

Of the studies that addressed the impact of COVID-19 related anxiety for non-special populations, several studies established an association between the COVID-19 pandemic and rates of anxiety among adolescents [16,17,18,24,28,30]. One study in China [28] identified that low support (Odds Ratio [*OR*] = 3.18, 95% Confidence Intervals [*CI*]: 2.54 to 3.98) and medium support (*OR* = 2.19, 95% *CI*: 1.94 to 2.48) increased the likelihood of anxiety. However, Chen et al. [19] did not identify a significant correlation between the COVID-19 and anxiety among adolescents.

### 3.2. Depression

Five studies (31%) addressed depression among non-special populations and identified an association between the pandemic and depression [17,25,27,28,30]. One study in China conducted by Duan et al. [17] identified an association between depression and COVID-19 related addiction in using social media, such as smartphone addiction (*OR* = 1.844, 95% *CI*: 1.29 to 2.811), and internet addiction (*OR* = 3.107, 95% *CI*: 1.252 to 7.708). Guo et al. [23] identified an association between COVID-19 related stress and depression (*β* = 0.33, *t* = 11.49, *p* < 0.001). However, the study conducted by Chen et al. in China [19] failed to establish a significant correlation between depression and COVID-19 related factors. 

### 3.3. Other Psychological Disorders/Distress

Of the studies, several considered various other forms of psychological disorders and other forms of psychological distress, such as burdensomeness, belongingness, psychological distress, stress and trauma, and drug abuse. One study in the United States [16] revealed that specific motivation to practice social distancing led to burdensomeness and belongingness among adolescents. Another study in the Philippines [27] identified that 16.3% of respondents experienced psychological impairments as moderate or severe due to the pandemic. A Chinese study by Zhang et al. [30] found that negative coping skills are risk factors for stress and trauma among junior high and high school students. A Canadian study [31] recognized the increased frequency of drug usage, such as alcohol and cannabis among adolescents during the pandemic.

### 3.4. Special Populations

Special populations among adolescents were assessed in several studies. These special population included Lesbian, Gay, Bisexual, Transgender, and Queer (LGBTQ) adolescents, adolescents diagnosed with anorexia nervosa, pre-pandemic maltreated adolescents, seniors, females, and adolescents diagnosed with obsessive-compulsive disorder (OCD). Through 31 synchronous text-based chats, LGBTQ adolescents expressed difficulty maintaining mental health wellness due to being forced to stay at home with unsupportive family members, and due to the lack of socialization that helps with identity [22]. Adolescents diagnosed with anorexia nervosa reported 70% increases in poor eating habits and increases in thoughts associated with eating disorders [21]. Pre-pandemic maltreated adolescents experienced higher rates of post-traumatic stress disorder (PTSD) (effect size beta = 0.16~0.27) and higher rates of anxiety (effect size beta = 0.32~0.47) [23]. Of the two studies [24,26] that specifically evaluated seniors, both studies established associations between poor mental health outcomes, such as psychological issues and anxiety, and factors related to the COVID-19 pandemic. Both studies that discussed gender-related data [26,27] established higher rates of COVID-19 related anxiety among females. The study [29] that evaluated various groups of youth, including adolescents that were diagnosed with OCD, established a worsening of symptoms (44.6%) for patients, who completed primary treatment and for patients, who were currently in psychiatric treatment. 

### 3.5. Benefits

Of the 16 studies in this review, one study in China [24] identified both a negative and a positive impact between COVID-19 related factors, such as home quarantining and parent–child discussions with mental health outcomes. The positive benefits were related with coming in closer and having more family discussions between parents and their children during home quarantining. 

## 4. Discussion

### 4.1. Impact on Mental Health

In this systematic review, there was conclusive evidence to support the potential negative impact of the pandemic on adolescent mental health. The stressors and motivations to practice social distancing due to the COVID-19 pandemic seem to be difficult for adolescents to process, which results in poor mental health outcomes [16]. The inefficient ability to process difficult circumstances, such as the pandemic are due to negative coping skills, which are risk factors for depression, stress and trauma among various ages of adolescents [30]. The lack of positive coping skills among adolescents is not unusual because adolescent must be provided with the tools to cope in order to be resilient and mentally well during periods of adjustments. However, the exposure to and practice of positive coping skills can lead to mentally well adolescents, who can easily adjust to rapid changes. 

Social support was another major factor identified in this review in determining the mental sustainability of adolescents during periods of crisis, such as the pandemic. Adolescents had perceived high rates of low to moderate social support during the pandemic, which contributed to increases in anxiety and depression [28]. Despite the concept of social support being expressed from the point of view of adolescents, which may or may not be bias, adolescents are still experiencing authentic forms of psychological challenges, such as anxiety and depression associated with the lack of social support and the pandemic. Due to these challenges, it is imperative that support be greatly implemented in homes. Studies have shown that the implementation of social support leads to positive mental health outcomes [28].

Addiction is another concern for adolescent mental health during the pandemic. Due to stay-at home orders, school closures, or new at home learning methods, students are seeking ways to connect and to socialize in manners that may not be productive for optimal health. The result of these behaviors are smartphone addiction and internet addiction. Both forms of addiction lead to poor mental health outcomes for adolescents. 

Despite being forced to remain at home, adolescents were identified as still engaging in drug use during the pandemic. Rather adolescents were increasing their use of alcohol and cannabis during the pandemic with 49.3% engaging in drug use alone [30]. An earlier study [32] showed the link between the lack of positive coping skills and the possible neurobiological pathways that may associate stress with the craving for drug use. 

### 4.2. Impact on Mental Health of Special Populations

As the non-special populations of adolescents experience mental health challenges, the special populations are experiencing a worsening of conditions, or experiences during the pandemic, which are a result of physical and psychologically COVID-19 related factors. These adolescents are forced to deal with unsupportive families due to sexual orientation, with the lack of social and medical support in connection to various pre-pandemic disorders, with an added stress concerning their futures, and with dealing with new and old stress from previous traumas [21,22,23,26,29,33]. These groups of adolescents have always experienced more challenges, but the pandemic has significantly affected the quality of life of these adolescents. 

## 5. Conclusions and Recommendations

Whether in the U.S. or abroad, the COVID-19 pandemic has impacted adolescent mental health. Stressful life events, extended home confinement, worry, overuse of the internet and social media are factors that could influence the mental health of adolescents during this pandemic. Adolescents from across the world face mental challenges due to COVID-19. Despite the uncertainty of the current crisis, it is important that adolescents receive the physical and mental care that they need to develop, to grow, and to thrive. Therefore, it is important to seek and to use all of the available resources and therapies to help adolescents mediate the adjustments caused by the pandemic. More research is needed on the improvement of adolescent mental health during COVID-19 and similar disasters. Among many interventions, more emphasis may be suggested on the global implementation of telemedicine to address the psychological needs of adolescents [34]. 

## Figures and Tables

**Table 1:** Inclusion and Exclusion Criteria.

Inclusion Criteria	Exclusion Criteria
Quantitative studies	Studies that were not in English
Human studies	Studies that only involved ages 18 and older
Scholarly papers	Review articles
Age group: 13–17	Not human studies
Mental health illnesses	Human Immunodeficiency Virus (HIV) and Autism Spectrum Disorder (ASD)
Research associated with COVID-19	

**Table 2:** Research Thread for all Databases.

Search Strategies	No. of Studies Available
Search terms used: ‘mental health’ OR ‘mental illnesses’ OR ‘mental disorder’ OR ‘psychiatric illness’ OR ‘anxiety’ OR ‘depression’ OR ‘COVID 19’ OR ‘coronavirus’ OR ‘2019-ncov’ OR ‘sars-cov-2’ OR ‘cov-19’ OR ‘adolescent’ OR ‘teenager’	1201
Total number of studies excluded based on eligibility criteria	1155
Total number of studies excluded because either they were review articles or they did not provide full articles	30
Total number of studies accepted and reviewed	16

**Table 3:** Impact of COVID-19 on adolescent mental health.

Author [Ref]	Major Findings	Impact on Mental Health	Quality Appraisal (Out of 4)	Country of Study
Oosterhoff et al., 2020 [16]	*n* = 683; specific motivations to implement social distancing practices are associated with anxiety, depression, burdensomeness and belongingness	Negative	4	United States
Duan et al., 2020 [17]	*n* = 3254; 22.8% respondents suffering from depression; certain factors were associated with depression and anxiety caused by COVID-19 related consequences	Negative	3	China
McElroy et al., 2020 [18]	*n* = 698; COVID-19 related anxiety validated during the first 6 weeks of the country’s lockdown	Negative	3	United Kingdom
Chen et al., 2020 [19]	*n* = 693; no significant correlation was established between anxiety or depression and the COVID-19 pandemic.	No	3	China
Isumi et al., 2020 [20]	*n* = 2022; no significant change in suicide rates between March 2020–May 2020 and 2018–2019	No	2	Japan
Schlegl et al., 2020 [21]	*n* = 159; approximately 70% of respondents reported issues with eating during the pandemic.	Negative	1	Germany
Fish et al., 2020 [22]	*n* = 159; LGBTQ youth have difficulty sustaining mental health wellness due to being isolated with unsupportive parents, and loss of identity due to lack of in person social engagement.	Negative	0	United States
Guo et al., 2020 [23]	*n* = 6196; pre-pandemic maltreatment and pandemic distress was associated with higher rates of anxiety and PTSD.	Negative	4	China
Tang et al., 2021 [24]	*n* = 4342; senior grades were at a greater risk of poor mental health outcomes, such as anxiety (24.9%), depression (19.7%), and stress (15.2%) due to the pandemic. Children and adolescents did exhibit benefits from home quarantining and parent–child discussions.	Negative, as well as Positive	4	China
Ellis et al., 2020 [25]	*n* = 1054; stress related to the COVID-19 pandemic was associated with higher rates of depression.	Negative	3	Canada
Qi et al., 2020 [26]	*n* = 9554; seniors and girls had higher prevalence of anxiety due to COVID-19 related factors.	Negative	4	China
Tee et al., 2020 [27]	*n* = 1879; 16.3% were impacted by psychological distress and16.9% were impacted by depression due to COVID-19 related factors. Females had higher rates of psychological distress, anxiety, and depression due to the pandemic.	Negative	4	Philippines
Qi et al., 2020 [28]	*n* = 7202; 70% of adolescents experience medium social support and 5.4% experience low social support. Low and medium social support is associated with 2–4-fold increase in depression or anxiety.	Negative	4	China
Nissen et al., 2020 [29]	*n* = 102; 44.6% of children, adolescents, and young adults with obsessive compulsive disorder exhibit a worsening of symptoms due to the pandemic.	Negative	2	Denmark
Zhang et al., 2020 [30]	*n* = 1025; negative coping with changes associated with the COVID-19 pandemic are risk factors for anxiety, stress, depression, and trauma related distress among junior high school and high school students.	Negative	4	China
Dumas et al., 2020 [31]	*n* = 1054; the pandemic was associated with an increase in the frequency of both alcohol and cannabis use among adolescents.	Negative	3	Canada

## Data Availability

Not applicable.
